# Aging in the sebaceous gland

**DOI:** 10.3389/fcell.2022.909694

**Published:** 2022-08-17

**Authors:** Xiaoxiao Hou, Ziyu Wei, Christos C Zouboulis, Qiang Ju

**Affiliations:** ^1^ Department of Dermatology, Renji Hospital, School of Medicine, Shanghai Jiaotong University, Shanghai, China; ^2^ Departments of Dermatology, Venereology, Allergology and Immunology, Dessau Medical Center, Brandenburg Medical School Theodor Fontane and Faculty of Health Sciences Brandenburg, Dessau, Germany; ^3^ Berlin Brandenburg Center for Regenerative Therapies, Charite Universitatsmedizin Berlin, Berlin, Germany; ^4^ Genetic Skin Disease Center, Jiangsu Key Laboratory of Molecular Biology for Skin Diseases and STIs, Institute of Dermatology, Chinese Academy of Medical Sciences and Peking Union Medical College, Nanjing, China

**Keywords:** aging, sebaceous gland, differentiation, hyperplasia, stem cell

## Abstract

Sebaceous glands (SGs) originate from hair follicular stem cells and secrete lipids to lubricate the skin. The coordinated effects of intrinsic and extrinsic aging factors generate degradation of SGs at a late age. Senescence of SGs could be a mirror of the late aging of both the human body and skin. The procedure of SG aging goes over an initial SG hyperplasia at light-exposed skin areas to end with SG atrophy, decreased sebum secretion, and altered sebum composition, which is related to skin dryness, lack of brightness, xerosis, roughness, desquamation, and pruritus. During differentiation and aging of SGs, many signaling pathways, such as Wnt/β-catenin, c-Myc, aryl hydrocarbon receptor (AhR), and p53 pathways, are involved. Random processes lead to random cell and DNA damage due to the production of free radicals during the lifespan and neuroendocrine system alterations. Extrinsic factors include sunlight exposure (photoaging), environmental pollution, and cigarette smoking, which can directly activate signaling pathways, such as Wnt/β-catenin, Notch, AhR, and p53 pathways, and are probably associated with the de-differentiation and hyperplasia of SGs, or indirectly activate the abovementioned signaling pathways by elevating the inflammation level. The production of ROS during intrinsic SG aging is less, the signaling pathways are activated slowly and mildly, and sebocytes are still differentiated, yet terminal differentiation is not completed. With extrinsic factors, relevant signaling pathways are activated rapidly and fiercely, thus inhibiting the differentiation of progenitor sebocytes and even inducing the differentiation of progenitor sebocytes into keratinocytes. The management of SG aging is also mentioned.

## Introduction

With the development of the industrialized society, more and more people are concerned about skin aging. Due to endogenous and exogenous (mostly sun exposure) factors, the thickening of the stratum corneum, xerosis, wrinkles, and abnormal pigmentation occur. Several studies have elaborated on epidermal and dermal aging; however, the aging of sebaceous glands (SGs) has barely been studied (Zouboulis et al., 2016). Aging of SGs, especially in the light-exposed areas, starts with SG hyperplasia, followed by atrophy, decreased sebum secretion, and occasionally the development of SG carcinoma. In this review, we illustrate SG aging from the aspects of SG alterations, molecular signaling pathway modifications with aging, the multiple causes of SG aging, and the manifestations and treatment of SG aging disorders.

## Stem cells, development, and differentiation of sebaceous glands

**FIGURE 1:**
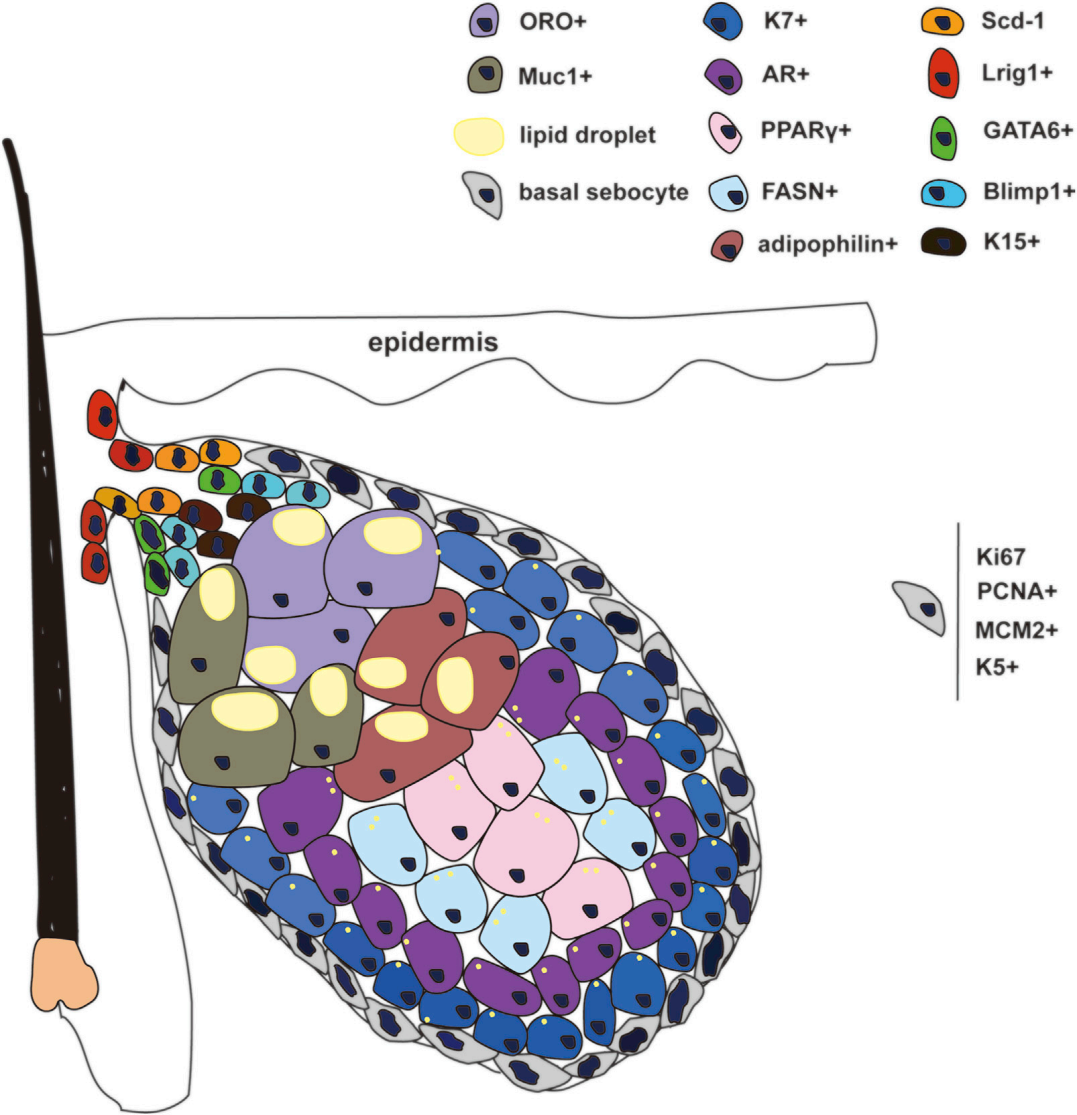
Different stages of cell pools and corresponding biomarkers in SG. Lrig1+, Scd-1, K15+, and GATA6+ cells represent the progenitor cells around the gland duct. Basal sebocytes can proliferate and differentiate. K7+ and AR+ cells represent the early differentiated sebocytes. PPARγ+ and FASN+ represent the differentiated sebocytes in the middle stage. ORO+ and Muc1+, and Adipophilin+ cells represent the terminally differentiated sebocytes.

Embryologically, the epithelium and its appendages develop from the ectoderm. Stem cells of the ectoderm differentiate into the interfollicular epidermis (IFE), hair follicles (HFs), sweat glands, and SGs under modulator signaling pathways, including Wnt, ectodysplasin A receptor (EDAR), bone morphogenetic protein (Bmp), and Hedgehog pathways (Schmidt-Ullrich and Paus, 2005; Duverger and Morasso, 2014; Saxena et al., 2019; Sennett et al., 2015). As part of the pilosebaceous unit, the development of SGs is closely associated with the formation of HFs. The initiation of SG development occurs in the upper region of the HF (Paus et al., 1999). During the development of SGs, first sebocytes differentiate from Lrig1+ HF stem cells which migrate to the distal HF epithelium close to the IFE (Figure 1). Meanwhile, the expression of stearoyl CoA desaturase 1 (SCD1) is detected concomitantly with the emergence of first sebocytes (Frances and Niemann, 2012). Lrig1 expression disappears once SCD1 is expressed in the SG progenitor cells. One cluster of SCD1-positive cells proceeds to the formation of two individual glands, and mature Lrig1-negative sebocytes are surrounded by Lrig1-positive stem cells. Those SCD1+ SG progenitor cells progress to proliferating basal cells anchored to the SG basement membrane. According to the results of Andersen et al. (2019), these progenitor cells undergo a defined process of random cell division and differentiation, which appears uncorrelated with the fate selection of neighboring cells, resulting in variable-sized SGs. Such a conclusion is opposed to the previous assumption that progenitor cells at the top of the gland replenish cells lost by differentiation at the basement membrane (Horsley et al., 2006). In the initial phase, B lymphocyte-induced maturation protein 1 (Blimp1)-positive cells represent a resident population of early differentiated sebocytes in mice as an intermediate stage between the progenitor and differentiated sebocytes, regulating the size and activity of SGs (Horsley et al., 2006; Kretzschmar et al., 2014). However, further studies have shown that Blimp1 is a terminal differentiation marker in human SGs (Magnusdottir et al., 2007). During the formation of SGs, keratin 15-positive cells are seen at the apical part of the SG, possibly representing SG precursors (Eisinger et al., 2010). The cells located at the basement membrane are positive for Ki67 (Andersen et al., 2019), proliferating cell nuclear antigen (PCNA) (Cottle et al., 2013), MCM2, and keratin 5 (Feldman et al., 2019). Basal sebocytes express the highest level of MYC in the SG. During maturation, MYC expression decreases, and SG proliferative cells progressively migrate and differentiate into the inner mass, from an early stage over middle stage to terminal differentiation, accumulating lipid droplets and eventually bursting to release lipids into the sebaceous duct. The early-stage differentiation markers are keratin 7 (K7) (de Bengy et al., 2019) and androgen receptor (AR) (Cottle et al., 2013). AR is highly expressed in the middle stage as well, and peroxisome proliferator-activated receptor gamma (PPARγ) and fatty acid synthase (FASN) are regarded as markers of middle-stage differentiation (Cottle et al., 2013). Terminally differentiated and mature sebocytes are oil red O (Feldman et al., 2019), melanocortin 5 receptor (MC5R), and mucin 1 (MUC-1) (Hinde et al., 2013; de Bengy et al., 2019), also known as the epithelial membrane antigen (EMA) and are adipophilin-positive (Hinde et al., 2013). Remarkably, K7 and MUC-1 are sebaceous markers in humans but not in murine SGs (Hinde et al., 2013). Homeostasis of SGs is maintained by the constant differentiation of sebocyte progenitor cells. Along with aging, progenitor cells are affected, and the SG differentiation was depleted (Zouboulis et al., 2008). Ki67 showed reduced expression in aged HFs in both human and mouse skin, revealing the diminished proliferation and regeneration of HFs (Chang et al., 2005; Ge et al., 2020). The protein level of PPARγ was found to be significantly downregulated in sebaceous glands based on the research that included 42 young and old human individuals (Elewa et al., 2015), indicating the reduced differentiation of sebocytes. However, in a mouse study, the activity of PPARγ and lipogenic genes such as acetyl-CoA carboxylase (Acc), fatty acid synthase (Fas), stearoyl-CoA desaturase 1 (Scd1), and sterol regulatory element-binding protein 1 (Srebp1) were elevated in an aging mouse model with chronic activation of p53 (Kim et al., 2014).

## Molecular variations associated with sebaceous gland aging

### Wnt/β-catenin signaling pathway

**FIGURE 2:**
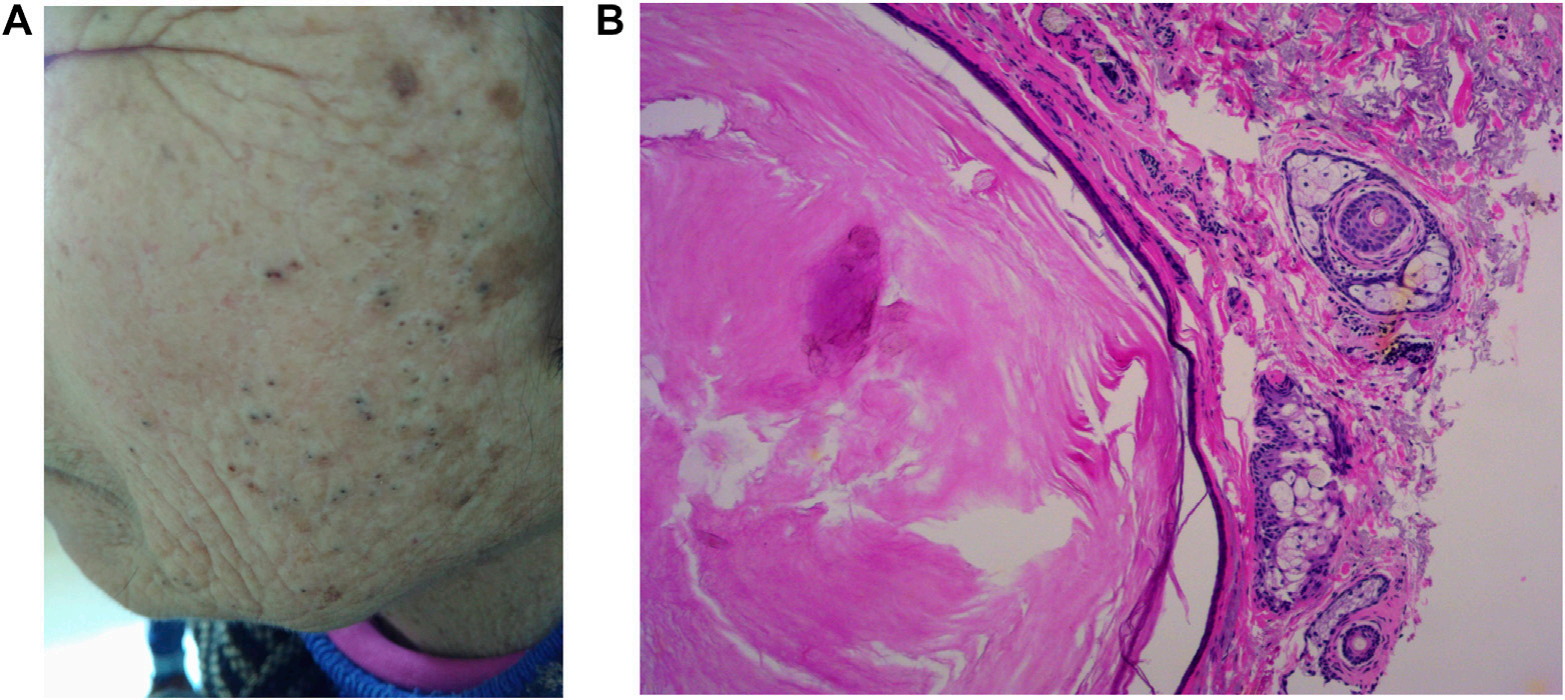
Favre–Racouchot disease (FRD) manifests as cutaneous atrophy and elastosis with keratinization of the pilosebaceous follicle and comedone formation and mainly affects the skin which is greatly exposed to sunlight **(A)**. Histology shows atrophic and keratinized SGs **(B)**.

The Wnt/β-catenin pathway, an important pathway in regulating epidermal differentiation, increases the expressions of involucrin and cornifin in SGs, reduces the number of terminally differentiated sebocytes, downregulates sebum secretion, and is related to epidermal cyst formation (Lo Celso et al., 2008; Shang et al., 2021). Loss of β-catenin in mouse epidermis leads to the enlargement of SGs (Niemann et al., 2002; Lien et al., 2014). Furthermore, AR activation was verified to reduce β-catenin-dependent transcription in SZ95 sebocytes and induce sebocyte differentiation (Ebling et al., 1969; Rosignoli et al., 2003). With aging, the level of serum AR is downregulated, and the inhibition of the Wnt/β-catenin signaling pathway is reduced, resulting in reduced SG differentiation and decreased lipid secretion, turning to the hyperplasia of SG and the formation of epidermoma (Ceruti et al., 2018). This is similar to what we observed clinically in solar elastosis comedones (literally epidermomas) (Figure 2), which develop after prolonged sun exposure (Patterson et al., 2004).

### Transforming growth factor-β

Transforming growth factor- (TGF-β) levels increase in dermal fibroblasts with aging (Gunin and Golubtzova, 2019). Interestingly, the significant activation of the TGF-β/Smad pathway in mouse skin-derived precursor supernatant after ultraviolet B (UVB) irradiation could alleviate the UVB irradiation damage (Li et al., 2020). This indicates that TGF-β may be an aging skin marker (Gunin and Golubtzova, 2019). Activation of the TGF-β signaling pathway has been found to downregulate sebocyte differentiation markers, such as fatty acid desaturase 2 (FADS2) and PPARγ, inhibit sebum secretion, and maintain the undifferentiated state of sebocytes (McNairn et al., 2013).

However, in fibroblasts, TGF-β was regarded as a rejuvenation marker during skin aging since it is a major regulator of the extracellular matrix, and reduction of TGF-β was involved in the degradation of collagen and elastin fibers. In aged skin, activator protein-1 (AP-1) inhibits the TGF-β signaling pathway in fibroblasts and decreased the synthesis of collagen (Fisher et al., 2016). UV induced inhibition of the TGF-β signaling pathway by downregulating the TGF-β receptor type II (TbRII) and over-expressing Smad7 in human skin epidermis (Han et al., 2005).

### p53

It has been demonstrated in several studies that the activation of p53 results in accelerated aging phenotypes in mice models (Tyner et al., 2002; Maier et al., 2004; Gannon et al., 2011), showing slow hair follicle cycling, epidermis thinning, reduced wound healing, and reduction of subcutaneous adipose lipid. Chronic activation of p53 can also lead to a decrease of Blimp1-positive sebocytes (sebaceous gland progenitor cells). Activation of p53 depletes the differentiation of sebaceous progenitor cells by activating PPARγ, resulting in the deplenishment of sebaceous progenitor cells, which in turn causes the atrophy of the entire sebaceous gland (Kim et al., 2014). It has also been reported that activation of p53 can inhibit c-MYC-induced sebaceous gland differentiation (Cottle et al., 2013) and attenuate the expression of insulin growth factor-1 receptor (IGF1R) (Werner et al., 1996) and AR (Shenk et al., 2001; Melnik, 2017), thus inhibiting the differentiation of sebocytes by suppressing the transactivation of PPARγ. In addition, p53 is mutated in 2/3 of sebaceous carcinomas (Kiyosaki et al., 2010), which is another manifestation of SG senescence.

### Aryl hydrocarbon receptor

Environmental pollutants are believed to induce a range of skin conditions, including skin aging. Since they are natural ligands of the aryl hydrocarbon receptor (AhR), they usually disturb cell differentiation and lipogenesis. AhR signaling mediates cell apoptosis, oxidative stress, hyperpigmentation, and subcellular organelle dysfunction induced by particulate matter (PM) 2.5 in HaCaT keratinocytes (Piao et al., 2018; Shi et al., 2021). Correspondingly, Liu et al. have shown that a standard reference material of air pollution PM induced human skin keratinocyte and dermal fibroblast aging through cell growth inhibition and cell arrest, which could cause skin barrier damage and collagen degradation. The translocation of AhR into the nucleus, ERK, and c-Jun activation and aging-related gene transcription play a vital role in the aging process (Qiao et al., 2017). AhR was found to be expressed in SGs and immortalized sebocytes (Ju et al., 2011). Activation of AhR inhibits lipogenesis and alters sebocyte differentiation by reversing the differentiation lineage toward keratinocytes (Hu et al., 2016). Therefore, reduced numbers of terminally differentiated sebocytes and reduced sebum secretion occur. AhR was proven to modulate peptidoglycan (PGN)-induced expressions of tumor necrosis factor (TNF)-α and interleukin (IL)-8 in human SZ95 sebocytes, which intensified the inflammatory signaling in SGs (Hou et al., 2019). Elevated inflammation plays a vital role in skin aging. In general, sustained activation of AhR may lead to SG aging in terms of cell development and inflammation.

### c-Myc

As a marker of basal proliferative sebocytes, c-Myc expression decreases along with the differentiation of sebocytes. Low levels of c-Myc activate AR, inhibit p53 activation, and promote SG differentiation and enlargement. High c-Myc activity induces p53 activation, thereby leading to SG proliferation and hyperplasia by blocking AR signaling (Lo Celso et al., 2008; Berta et al., 2010; Cottle et al., 2013). c-Myc mRNA and protein levels increased in SZ95 sebocytes incubated with elderly (60-year-old) female hormones for 5 days compared to sebocytes maintained in young (20-year-old) female hormones (Makrantonaki et al., 2006), which indicate that the differentiation ability decreased in the SG along with aging.

### Hedgehog

Upregulation of the Hedgehog (Hh) pathway stimulates the proliferation of undifferentiated sebocytes, and there are crosstalks between Hh and Wnt-β-catenin signaling pathways (Niemann et al., 2003). The expression trend of Hh was consistent with that of β-catenin (Gat et al., 1998; Huelsken et al., 2001). Using the Hh inhibitor could reduce the cystic structures caused by aberrant activation of the Wnt-β-catenin signaling pathway (Shang et al., 2021). The Hh pathway also plays a vital role in basal cell carcinoma pathogenesis (Fania et al., 2020).

### Notch

The Notch pathway has been reported to be involved in a variety of adult aging-related diseases, such as Alzheimer’s disease, and cerebrovascular and cardiovascular diseases (Balistreri et al., 2016). *NOTCH2* was also found to be downregulated in aging sebocytes (Makrantonaki et al., 2006). Expressions of AR and PPARγ, as markers of early sebocyte differentiation, were detected unchanged even when the Notch pathway was knocked out; however, the expression of the terminal differentiation marker FASN was completely downregulated. Interestingly, SG cells rested at a stage of primary differentiation without progressing to full differentiation. Consequently, the accumulation of lipids started but stalled (Veniaminova et al., 2019).

Excluding the specific SG markers, genes involved in mitochondrial function, oxidative damage, and stress response showed altered expression in hormonally aged sebocytes, a fact that might lead to an increase in the accumulation of free radicals (Makrantonaki et al., 2006). Genes involved in the ubiquitin-proteosome pathway were downregulated, resulting in the accumulation of highly misfolded and damaged proteins. The expression of genes involved in cholesterol and fatty acid biosynthesis declined, contributing to the decrease in sebum amounts (Makrantonaki et al., 2006).

## Changes in clinical features of sebaceous glands with aging

### Sebum changes with aging

Sebaceous lipids are ubiquitously synthesized from sebocytes and secreted together with cell debris as sebum, contributing to ultraviolet protection, antioxidation, compound absorption, antibacterial effects, and skin hydration to protect the human skin (Zouboulis et al., 2016). Sebum secretion is relatively low in children as the level of circulating androgens including testosterone, dehydroepiandroster one sulphate (DHEAs), and insulin growth factor-1 begins to increase with adrenarche and further during puberty. The onset of puberty is often accompanied by a marked physiological increase in sebum, which is an important factor in the pathophysiology of acne vulgaris (Rocha and Bagatin, 2018). In elderly males, sebum production remains almost unchanged compared with that of young males even at the age of 80, while the sebum content in women begins to decline with menopause (Pochi et al., 1979; Zouboulis et al., 2022). In a large Chinese cohort, the skin surface sebum content was measured, and it was found that there was a peak at around the age of 40 years in females and 50 years in males, which could be some race/ethnic disparities. Meanwhile, the sebum content on the forehead in both males and females was higher than that on the forearm, and the level of sebum in males was always higher than that in females in different age groups (Man et al., 2009).

In addition, substantial changes in sebum composition occur with aging. As early as 1972, Cotterill et al. (1972) reported no significant differences in the sum of the percentages of triglycerides (TG) and free fatty acids (FFA) among different ages or sex, while the degree of hydrolysis varied considerably with age. Squalene is an unsaturated hydrocarbon produced by human SGs, and its content reached a maximum between the ages of 20 and 40 years in males, thereafter decreasing in the 41–60 age group. In addition, wax ester secretion rates reached their highest at the age between 15 and 35 and appeared to decline continuously throughout the elderly age range (Jacobsen et al., 1985). It should also be mentioned that photoaging could cause a range of changes in sebum components. Kim et al. (2010) observed that the levels of TG and FFA were significantly decreased in the epidermis of photoaged or acutely ultraviolet (UV)-irradiated human skin. Furthermore, they also demonstrated that triolein reduced basal and UV-induced metalloproteinase-1 (MMP-1) mRNA expression in cultured human epidermal keratinocytes, while various lipid synthesis enzyme inhibitors increased the MMP-1 expression significantly in a dose-dependent manner, hinting that TG and FFA may play important roles in photoaging of the human skin. In addition, free radicals generated by UV light could induce oxidative stress and promote the formation of squalene hydroperoxide and then cause the thickening of the epidermal layers, forming many deep crests on the skin surface and ultimately leading to skin wrinkling and photoaging in the human skin (Gat et al., 1998; Niemann et al., 2003) and hairless mouse skin (Chiba et al., 1999; Chiba et al., 2003). Moreover, UVB radiation can also affect lipid levels and lipid profiles *in vitro* and *in vivo* (Akitomo et al., 2003; Sato et al., 2017). Clinical research comparing all the differences in the sebum composition at the same time of individuals of different ages is still lacking.

With aging, skin becomes dryer and characterized by a lack of brightness of the skin surface, roughness, xerosis, desquamation, and pruritus, which is related to the decrease in sebum secretion and the reduced levels of epidermal and sebaceous lipids with age (Balin and Pratt, 1989).

### Morphological and pathological changes of sebaceous glands with aging

The number of SGs basically remains unchanged, while the size of the SGs tends to initially increase with aging in the early stage (Plewig and Kligman, 1978; Fenske and Lober, 1986), especially in light-exposed skin (Zouboulis et al., 2016). However, in the late stage of aging or with excessive light exposure, the SGs would atrophy. Favre–Racouchot disease (FRD) is a typical disorder that mainly affects the elderly who are significantly exposed to sunlight. It manifests as cutaneous atrophy and elastosis with keratinization of the pilosebaceous follicle and the formation of pseudocomedones, which represent superficial epithelial tunnels (Helm, 1961; Patterson et al., 2004) (Figure 2A). The keratinization of the pilosebaceous follicle (Figure 2B) is assumed to be associated with the activation of Wnt/β-catenin, NOTCH, and p53 pathways, which leads to the proliferation and de-differentiation of sebocytes. There is probably a similarity in the pathogenesis of intrinsically aging-induced SG hyperplasia and photoaging-induced FRD.

**FIGURE 3:**
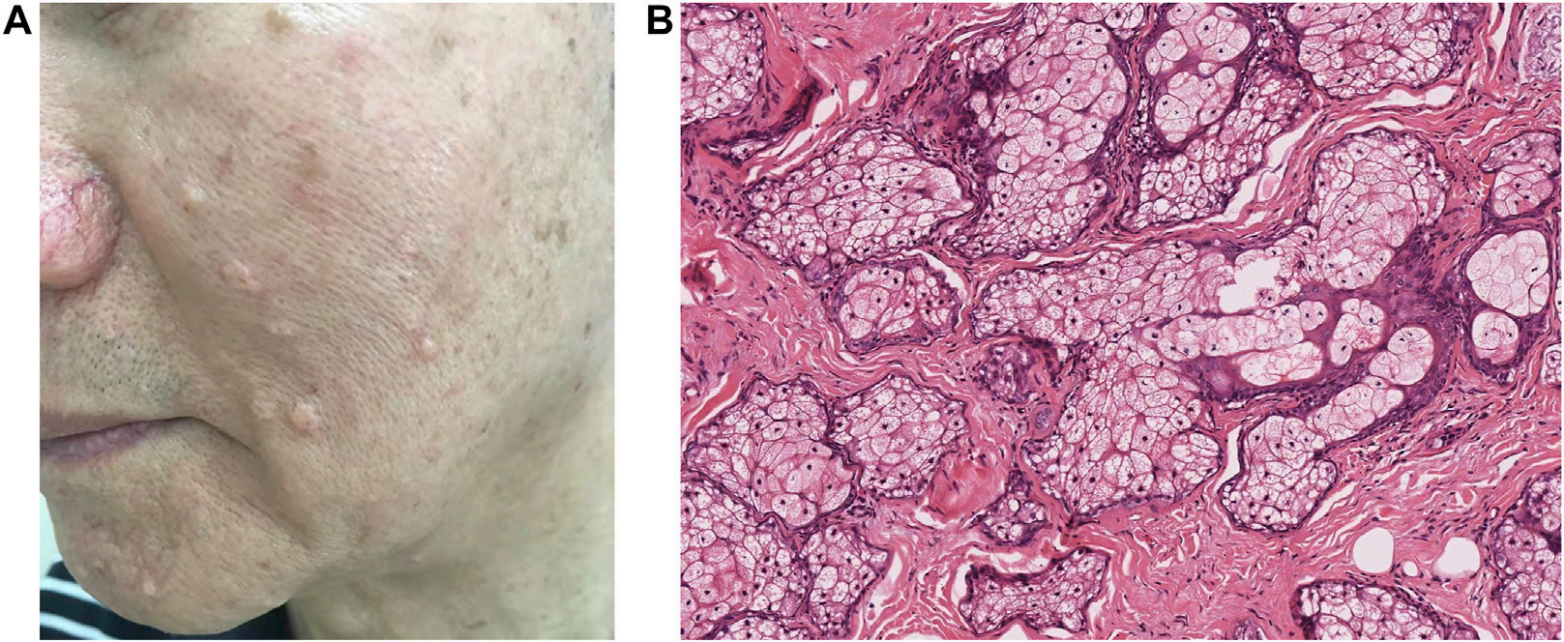
Skin-colored papules represent hyperplastic sebaceous glands disseminated on the face of an elderly patient. Clinically manifested as single or multiple pale yellow or skin color papules and nodules **(A)**, a great number of progenitor cells, and less mature sebocytes in histology **(B)**.

The incidence of SG hyperplasia is 1% among healthy people, while in patients undergoing heart transplantation and taking immunosuppressive medications, it is 16% (de Berker et al., 1996). SG hyperplasia develops mainly in patients above 50 years, and it is mostly seen in the forehead and cheeks of elderly people, which may be related to the exposure of chronic sun exposure (Zouboulis and Boschnakow, 2001), and is clinically manifested as single or multiple pale yellow or skin color papules and nodules with a diameter of about 1–5 mm (Figure 3).

It is believed that the occurrence of SG hyperplasia in the elderly may be related to the decrease in androgen levels, which reduces the cellular turnover of sebocytes and subsequently leads to compensatory hyperplasia of SGs (Plewig and Kligman, 1978; Pochi et al., 1979; Fenske and Lober, 1986). In addition, the hormonal influence of insulin, thyroid stimulating hormone, and hydrocortisone may also increase sebocyte proliferation and contribute to SG hyperplasia (Fabiola Farci, 2022). At the same time, UV, especially UVA, may also cause SG hyperplasia in elderly patients and also induce the secretion of inflammatory cytokines including interleukins IL-1β and IL-8 in human sebocytes *in vitro* (Lee et al., 2013). However, opposing opinions have also been presented that chronic solar exposure was not a likely cause of the occurrence of SG hyperplasia and senile pseudocomedones (Kumar and Marks, 1987). Further studies are required to investigate how androgens and UV radiation interact with cellular turnover and differentiation of sebocytes then leading to SG hyperplasia.

The histological examination shows that numerous SGs filled with mature sebocytes are diffusely distributed in the superficial dermis, and the lobules open in the center of the dilated SG duct. The presence of four or more sebaceous lobules around a hair follicle has been suggested as a diagnostic criterion (Fabiola Farci, 2022). The prominent mature sebocytes present a vacuolized morphology and are rich in lipid vesicles. The basement membrane of SGs in young people tends to be thick, while the rim of basal cells in the elderly is much thinner. In addition, their fibers in the upper dermis are no longer elastic and manifest as distorted, thicker, and coagulated (Montagna and Carlisle, 1990; Zouboulis and Boschnakow, 2001).

## Factors of sebaceous gland aging

Skin aging including SG aging can be classified as physiological (internal/chronological) aging and exogenous aging. The intrinsic factors that cause chronological aging include genetic, neuroendocrine system variation, and skin diseases.

**FIGURE 4:**
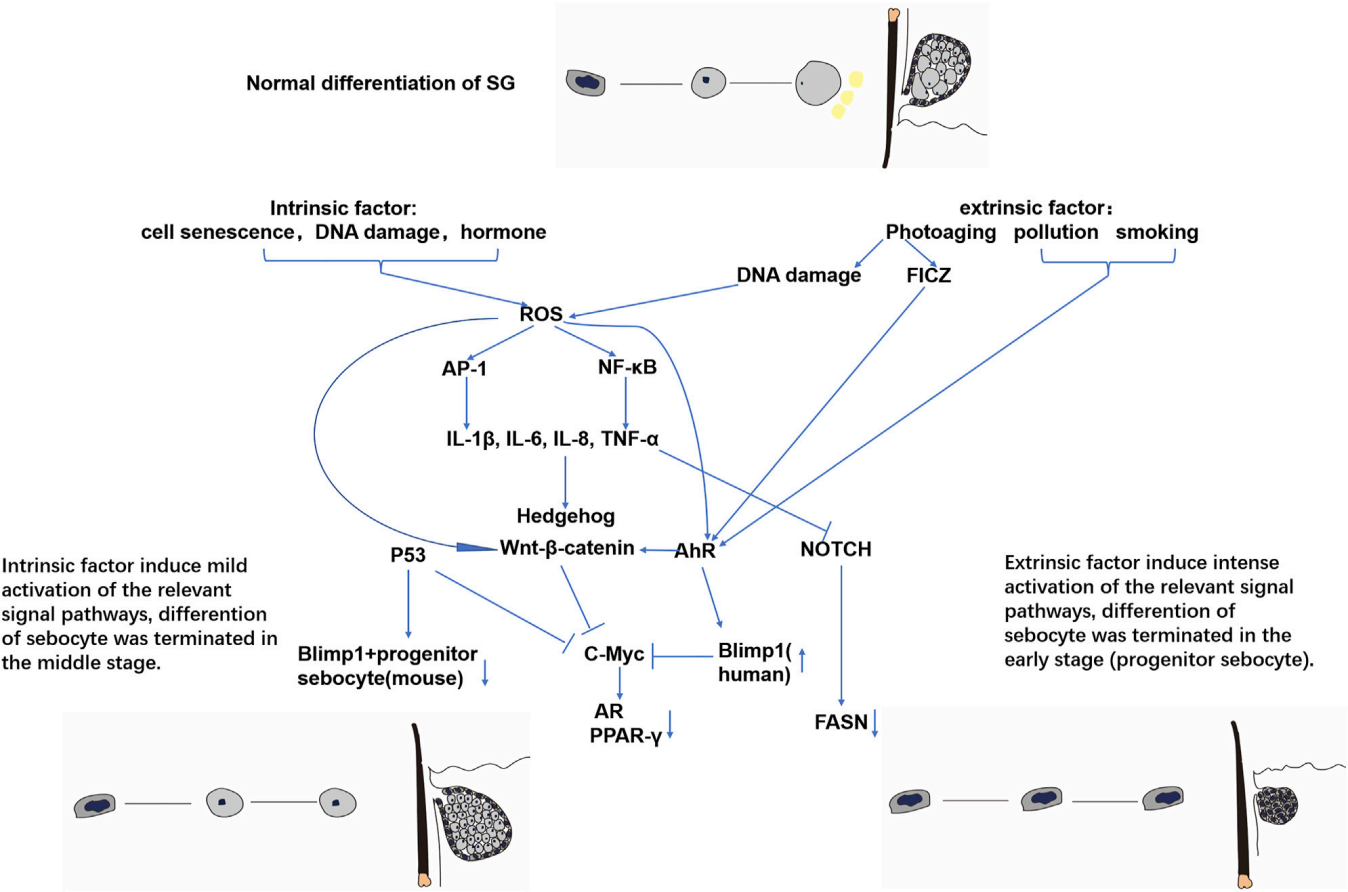
Possible molecular mechanisms of intrinsic and extrinsic SG aging.

Genetically, random processes lead to random cell senescence and DNA damage due to the production of free radicals, which also modify the inflammation status in the skin (Puizina-Ivic, 2008). Endogenous reactive oxygen species (ROS) are also heavily produced by mitochondria as they age (Chance et al., 1979). On the other hand, the neuroendocrine system varies along with the age, adrenal secretion of the steroid precursors dehydroepiandroster one (DHEA) and DHEAs, and hormones which are converted into androgen and estrogens and gradually decline over time (Herbert, 1995; Ferrari et al., 2000). (Figure 4).

Extrinsic factors, which might influence SGs' functions, include sunlight exposure (photoaging), pollution, smoking, and lifestyle factors such as diet, sleeping rhythm, and alcohol intake.

### Photoaging

UVC (100–290 nm) is mostly blocked by the ozone layer, while UVB only penetrates into the epidermis and causes skin pigmentation. It has also been involved in photocarcinogenesis and skin-associated immunosuppression (Gilchrest, 1996; Benjamin et al., 2008). UVA is known to penetrate the dermis and is acutely responsible for skin erythema and mostly chronic skin damage (Gilchrest, 1996). Cumulative UVA is absorbed by cellular chromophores and generates ROS, including superoxide anion, hydrogen peroxide, and singlet oxygen, which could induce transcription factor activator protein-1 (AP-1) and nuclear factor kappa-B (NF-κB). The activation of AP-1 leads to the elevated expression of metalloproteases (MMPs), which could degrade collagen I and III. The activation of NF-κB upregulates the expression of a series of proinflammatory cytokines including IL-1β, TNF-α, IL-6, and IL-8. ROS production activates the Wnt/β-catenin pathway during mesenchymal stem cell aging (Zhang et al., 2013). Further research about the exact mechanism between ROS and the Wnt/β-catenin pathway in sebocytes needs to be conducted. It can be reasonably assumed that ROS and chronic inflammation could lead to SG hyperplasia through the activation of Wnt/β-catenin, NOTCH, Hedgehog, and p53 pathways.

### Environmental pollution

An increasing number of studies have investigated the association between environmental pollution and skin aging (Li et al., 2015; Huls et al., 2016; Ding et al., 2017; Fuks et al., 2019). Pollutants include O_3_, PM, nitrogen oxide (NO_2_), cigarette smoke, and solid fuels, which can generate a substantial amount of polycyclic aromatic hydrocarbons (PAHs) and carbon monoxide (Vierkotter et al., 2010; Clark et al., 2013; Huls et al., 2016; Fuks et al., 2019). Mechanistically, these environmental pollutants generate free radicals on the skin including SGs, further activating nuclear factor erythroid-related factor 2 (Nrf2), AhR, AP-1, and NF-κB. On the other hand, 4-hydroxynonenal, the main product of oxidative stress after exposure to O_3_, cigarette smoke, and PM, could directly regulate the activity of Nrf2, AP-1, PPARs, and AhR. As we mentioned earlier, upregulation of AhR could further reduce the lipid secretion of SGs by promoting sebocyte differentiation into keratinocytes. The evaluated inflammation through activation of AP-1 and NF-κB can induce the keratinization and hyperplasia signaling pathways in SGs. In an own previous study, benzo(a)pyrene (BaP), a compound found in cigarette smoke (Ortiz and Grando, 2012), has been shown to stimulate the secretion of IL-6 and reduce lipogenesis in SZ95 sebocytes (Sheikh et al., 2016; Hu et al., 2016). 2,3,7,8-Tetrachlorodibenzodioxin (TCDD) is the most potent compound of PAHs and the classic agonist of AhR, and its accumulation in sebum results in de-differentiation of sebocytes and dermal cyst formation by inhibition of the c-Myc signaling pathway and upregulates the Wnt pathway (Kretzschmar et al., 2014). TCDD could also enhance TNF-α and IL-8 secretion in PGN-treated sebocytes as well (Hou et al., 2019), further reinforcing SG aging.

## Management of sebaceous gland aging

### Prevention

Skin aging is a dynamic, multifactorial process, and the evidence level of the management of SG aging is still lacking. Several topical skin care products have been introduced in the prevention and treatment of SG aging, including sunscreens, anti-oxidants, vitamin C, and vitamin E (Zouboulis and Makrantonaki, 2011). Topical use of vitamins C and E improved wrinkles, skin tone, and texture, indicating their anti-aging and brightening effects of skin (Rattanawiwatpong et al., 2020; Jagdeo et al., 2021). In addition, eating a diet that is high in vegetables and fruits and avoiding cigarette smoking and pollution should also be noted (Farage KWM and Maibach, 2017). Recently, some randomized controlled studies had demonstrated that daily almond consumption may reduce wrinkle severity and improve skin pigmentation in postmenopausal women (Foolad et al., 2019; Rybak et al., 2021).

### Treatment

Age-related natural hormone reduction is a common condition that can be typically treated with hormonal replacement therapy (HRT). Previous studies have confirmed that estrogen use was associated with a statistically significant decrease in the likelihood of senile dry skin and skin wrinkling (Dunn et al., 1997). Moreover, topically administered estradiol and methyl estradiolpropanoate (MEP) as in anti-aging cosmeceuticals with estrogen-like cutaneous effects have also been found to increase sebum levels and improve skin dryness in menopausal women (Callens et al., 1996; Draelos, 2018). Moreover, skin surface lipids have been shown to be increased in patients supplemented with both estrogen and progesterone, while estrogen alone has a sebum-suppressive action, hinting at the sebum secretion promoting effects of progesterone (Sator et al., 2001). Safety concerns have led to the application of HRT with bioidentical hormones at individualized doses tailored to each patient (Rosenthal et al., 2019). Apart from bioidentical hormones, newly discovered phytoestrogens from fermented soybean extracts have been found to improve skin hydration and viscoelasticity in rats without systemic toxicities (Rungseevijitprapa et al., 2021). However, it should be noted that HRT could increase the risk of breast, endometrial, and ovarian cancers, so the doses should be tailored to each patient (Rees, 2011). Further studies should be conducted to provide more evidence in the treatment of SG aging.

SG hyperplasia is a relatively benign disorder, which does not usually require treatment. However, skin biopsies should be performed to differentially diagnose non-melanoma skin cancer (Salim et al., 2006). In addition, treatment can be conducted when skin lesions are unsightly and cause psychological distress for patients. Several treatment options exist including cryosurgery (Ataş and Gönül, 2017), photodynamic therapy (Horio et al., 2003), laser treatment (argon, carbon dioxide, or pulsed-dye laser) (Aghassi et al., 2000; Simmons et al., 2015), cauterization or electrodesiccation shaving or excision (Bader and Scarborough, 2000), topical treatments with chloroacetic or trichloroacetic acid, and systemic treatment with isotretinoin (Farage KWM and Maibach, 2017).

## Summary

Research and discussions on skin aging focus on epidermal changes and degradation of dermal collagen. This review mainly discussed the alteration in lipid secretion and the changes in related molecular mechanisms of SGs in endogenous and exogenous aging. In the initial stage of aging, the differentiation of SGs is inhibited, and the proliferation is increased. Therefore, SGs show reduced lipid secretion and gland hyperplasia. In the late stage of aging with excessive photo exposure or environmental pollution, the over-activation of related molecular signaling pathways causes SG progenitor cells to differentiate into keratinocytes, which induces keratinization of pilosebaceous units, the most characteristic manifestation of Favre–Racouchot disease. There are still many unknown mechanisms in SG aging to be explored, and research needs to focus on the SG aging molecular mechanisms.
